# Optical Coherence Tomography Angiography (OCTA) in Multiple Sclerosis and Neuromyelitis Optica Spectrum Disorder

**DOI:** 10.3389/fneur.2020.604049

**Published:** 2020-12-10

**Authors:** Iris Kleerekooper, Sarah Houston, Adam M. Dubis, S. Anand Trip, Axel Petzold

**Affiliations:** ^1^Department of Neuro-Ophthalmology, Moorfields Eye Hospital, London, United Kingdom; ^2^Queen Square MS Centre, UCL Institute of Neurology and National Hospital for Neurology & Neurosurgery, London, United Kingdom; ^3^Institute of Ophthalmology, University College London, London, United Kingdom; ^4^National Institute for Health Research, Biomedical Resource Centre at University College London, Institute of Ophthalmology and Moorfields Eye Hospital National Health Service Trust, London, United Kingdom; ^5^Dutch Expertise Centre of Neuro-Ophthalmology, Amsterdam UMC, Amsterdam, Netherlands

**Keywords:** OCTA, OCT, multiple sclerosis, NMOSD, optic neuritis, microvascular

## Abstract

Vascular changes are increasingly recognized as important factors in the pathophysiology of neuroinflammatory disease, especially in multiple sclerosis (MS). The relatively novel technology of optical coherence tomography angiography (OCTA) images the retinal and choroidal vasculature non-invasively and in a depth-resolved manner. OCTA provides an alternative quantitative measure of retinal damage, by measuring vascular density instead of structural atrophy. Preliminary results suggest OCTA is sensitive to retinal damage in early disease stages, while also having less of a “floor-effect” compared with commonly used OCT metrics, meaning it can pick up further damage in a severely atrophied retina in later stages of disease. Furthermore, it may serve as a surrogate marker for vascular pathology in the central nervous system. Data to date consistently reveal lower densities of the retinal microvasculature in both MS and neuromyelitis optica spectrum disorder (NMOSD) compared with healthy controls, even in the absence of prior optic neuritis. Exploring the timing of vascular changes relative to structural atrophy may help answer important questions about the role of hypoperfusion in the pathophysiology of neuroinflammatory disease. Finally, qualitative characteristics of retinal microvasculature may help discriminate between different neuroinflammatory disorders. There are however still issues regarding image quality and development of standardized analysis methods before OCTA can be fully incorporated into clinical practice.

## Introduction

In addition to well-established immune mediated processes, vascular and metabolic factors are increasingly recognized to play important parts in the pathophysiology of neuroinflammatory disease, especially in multiple sclerosis (MS) (1). Optical coherence tomography angiography (OCTA) is a relatively novel technology that images the retinal and choroidal vasculature in a non-invasive and depth-resolved manner (2). OCTA technology is derived from additional processing of optical coherence tomography (OCT), a structural retinal imaging modality first used in 1991 that has since secured its place as a staple of neuro-ophthalmological clinical evaluation (3–5). Currently, some attention is diverted to the exciting potential that OCTA has in imaging the retinal vasculature using similar hardware and procedure.

Due to the design of the eye, the retina is one of the most easily accessible internal structures for imaging inside the human body. The retina is part of the central nervous system (CNS), as it contains the retinal nerve fiber layer (RNFL) and ganglion cell layer (GCL) which are formed of cells that relay visual information to the brain through the optic nerve (6, 7). Additionally, the anterior visual system is one of the most metabolically active structures in the human body and, accordingly, has a high perfusion rate which is rapidly adaptive to temporal changes in local demand (8–10).

The optic nerve is commonly injured in MS and neuromyelitis optica spectrum disorder (NMOSD) by optic neuritis (ON) (11–14). Demyelinating damage to the anterior visual system is virtually ubiquitous in postmortem studies of MS (15, 16). Thinning of the peripapillary RNFL (pRNFL) and GCL, as measured with OCT, have been shown to be markers of previous ON. However, retinal atrophy is also present in the absence of prior symptomatic ON and provides a valuable surrogate for more general, independent, CNS atrophy in neuroinflammatory disease (5, 11).

Even though immune mediated mechanisms are considered the most important drivers of neuroinflammatory disease, accumulating evidence indicates that CNS tissue energy failure, caused by hypoperfusion and hypoxia, is present in MS patients. This energy crisis may render neurons ineffective during an acute relapse and cause damage to oligodendrocytes which sets in action insidious processes of chronic demyelination and atrophy (1, 17). Neuropathologic studies of NMOSD affected tissue show that CNS lesions have a clear predilection for forming in perivascular locations (18). Furthermore, vessel walls in surrounding area are found to be fibrosed and hyalinised, a change in vessel morphology that can even be picked up on fundoscopy of NMOSD patients (19). In the acute clinical setting it can be difficult to differentiate between ON associated with MS and NMOSD, while the two diseases generally warrant different treatment decisions (13). Qualitative differences in vascular morphology picked up with OCTA may be diagnostically valuable in acute ON patients when differentiating between these conditions.

OCTA may be implemented in several interesting ways to advance the understanding of neuroinflammatory disease. Studies investigating OCTA in neuroinflammatory disease found that its findings are usually highly correlated to measurements of retinal layer thickness performed with the more consolidated technique of OCT (20–22). Therefore, OCTA can be an alternative quantitative measure of damage to the retina and optic nerve, as atrophied tissue requires less blood supply (20). Preliminary results in glaucoma research suggest that OCTA is sensitive to retinal damage in the earliest disease stages, while also being able to detect further damage in a severely atrophied retina, due to its low “floor-effect” (23, 24). In early-stage glaucoma, vascular density loss is faster compared with GCL thinning (25). Furthermore, another study concluded that the OCTA may be a promising tool to follow-up patients with late-stage glaucoma, as the measurement floor is lower compared with commonly used OCT metrics (26).

Additionally, OCTA may provide an avenue for investigations into vascular changes in the CNS. Preliminary OCTA research shows the retinal microvasculature is affected in both MS and NMOSD, even in eyes unaffected by ON and in some cases in the absence of structural retinal atrophy (20, 27). Investigating when vascular changes occur relative to thinning of retinal layers in neuroinflammatory disease may help answer important questions about the role of hypoperfusion in their pathophysiology (10). Finally, certain qualitative characteristics of the retinal microvasculature on OCTA may provide information for diagnostic discrimination between different causes of ON (28), similar to how the “central vein sign” on high-field MRI is a valuable diagnostic marker for MS (29). However, issues remain regarding image quality and the lack of standardization in image collection and analysis, which must be addressed before OCTA can be fully incorporated into clinical practice similar to what a network approach achieved for OCT (30).

In this review, we will discuss the potential value of OCTA in advancing research and clinical care in neuroinflammatory disease. First, we give an overview of the technical background of OCTA and the anatomy of retinal vasculature. Subsequently, we elaborate on the role of vascular alterations, particularly in the retina, in neuroinflammatory disease and we summarize research using OCTA in MS and NMOSD until now and finally, we discuss future prospects and the advantages as well as disadvantages of OCTA.

## Technical Background OCTA

OCT uses a technique that can be described as an optical analog to ultrasound-based imaging technology. OCT creates *in-vivo* structural images from biological tissue based on interferometry of low-coherence, near infrared light that is split into a reference arm, reflecting off a reference mirror, and a sample arm, reflecting from the investigated tissue. A strong interference occurs when both reflected beams recombine and a pattern of interference allows us to create a reflectivity profile, which is used to construct the OCT images (3). Currently, several generations of commercially available OCT devices are available and widely used.

**Figure 1:**
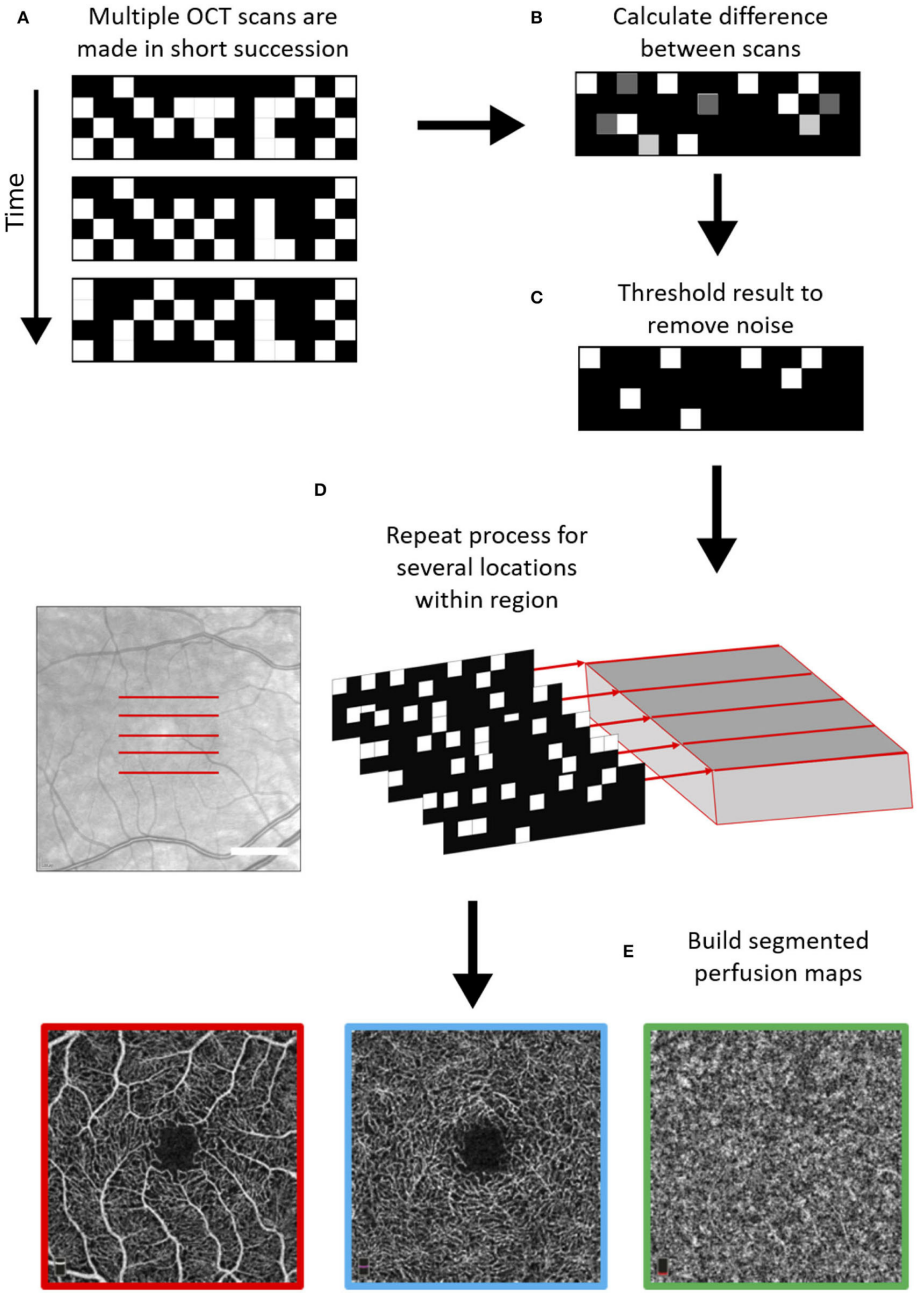
Schematic of general optical coherence tomography angiography (OCTA) scanning technique. First, multiple OCT scans are repeated with a high frequency at the same location **(A)**, and based on differences between these scans in the phase, amplitude, and/or intensity of the backscattered light, areas with moving particles are differentiated from static tissue **(B,C)**. This process is repeated over several locations **(D)** and using a segmentation protocol perfusion maps are created for the different vascular plexi **(E)**. The example scans shown below are the superficial vascular plexus (red), the deep vascular plexus (blue), and the choroid (green) as obtained with the Heidelberg Engineering OCT2 system.

OCTA expands on this technique by looking at temporal changes in the quality of backscattered light to distinguish locations of static tissues from blood flow. It detects blood vessels based on differences in amplitude, intensity, or phase variance between sequential OCT B-scans in the same position of the retina. Sequential B-scans are taken at the same transverse location and compared. The difference in signal between images reflects blood cells flowing through vessel lumen. Therefore, blood movement is used as an intrinsic contrast agent to create a vascular map (see Figure 1). Because of its reliance on picking up tiny temporal changes, a high sampling frequency is necessary to create sufficient quality OCTA images (31, 32). It is important to remember that as a result of this technique, there is a floor to detectable OCTA signal that is slightly higher than slowest physiological flow, although the minimal detectable flow speed is not known.

Scans are visualized as en-face images of individual vascular layers, segmented from the volumetric map. The retinal vascular plexi are usually segmented in OCTA as a superficial layer, which consists of the superficial vascular plexus, and a deep layer, which takes the inner and deep vascular plexi together. But importantly, not all OCTA systems use the same segmentation algorithm for the superficial and deep vascular layers (33). Anatomical constraints and offsets from structural boundaries may vary between instruments (Table 1) (33).

**Table 1:** OCTA platforms and segmentation of vascular plexi.

**Platform**	**Instrument specifics**	**Vascular plexus**	**Slab boundary**	**Anatomic basis**	**Offset, μm**
PLEX® Elite 9000 (Carl Zeiss Meditec)	Version 2018 v1.7 (Native seg); Version 2020 v2.1 (MLS)	Superficial (Native seg)	Top	ILM	0
			Bottom	IPL= ILM + 0.7·(OPL-ILM)	0
		Superficial (MLS)	Top	IML	0
			Bottom	IPL	−10
		Deep (Native seg)	Top	IPL	0
			Bottom	OPL (RPEFit – 110 μm)	0
		Deep (MLS)	Top	IPL	−10
			Bottom	OPL	0
CIRRUS™ HD-OCT 5000 with AngioPlex® (Carl Zeiss Meditec)	Version 9.5.0.8712; firmware 1.100.0.8; engine 6.5.0.8523	Superficial	Top	ILM	0
			Bottom	IPL= ILM + 0.7·(OPL-ILM)	0
		Deep	Top	IPL	0
			Bottom	OPL= RPEFit – 110 μm	0
RTVue XR Avanti (Optovue)	Version 2016.1.0.2	Superficial	Top	ILM	3
			Bottom	IPL	15
		Deep	Top	IPL	15
			Bottom	IPL	71
Triton DRI OCT (Topcon Medical Systems)	Version 10.07.003.03; IMAGEnet 6 version 1.14.8741	Superficial	Top	ILM	2.6
			Bottom	IPL/INL	15.6
		Deep	Top	IPL/INL	15.6
			Bottom	IPL/INL	70.2
Spectralis HRA OCTA (Heidelberg Engineering)	Version HEYEX V6.4a	Superficial	Top	ILM	0
			Bottom	IPL	0
		Deep	Top	IPL	0
			Bottom	IPL	0

*ILM, inner limiting membrane; IPL, inner plexiform layer; OPL, outer plexiform layer; RPE, retinal pigmented epithelium; INL, inner nuclear layer*.

OCTA images are generally analyzed by determining vessel density as the percentage of white pixels (representing vessels) in a segmented area. Although simple and easily implemented, this form of analysis is significantly influenced by the presence of artifacts, which are discussed later in this review. Most look at the vascular densities within a circular or square area around the fovea or optic disc. Densities of the macular SVP, DVP, and choroidal plexus are quantified separately in prespecified areas of investigation, generally a square of 3x3 or 6x6 mm. A separate outcome measure is the “foveal avascular zone” (FAZ), that estimates the size of the circular area in the center of the macula that has no SVP blood supply. The size and shape of the FAZ have both been used as an outcome, particularly in diabetes mellitus (DM) (34, 35). The FAZ has not yet been widely used as an outcome measure in neurological disorders.

A key feature of the healthy microvasculature is its fractal pattern, where shape of the vasculature is similar at every scale, fractal analysis has been gaining traction as an additional analysis tool (36, 37). This kind of analysis could be especially useful in cases of subtle microvascular change where there are no large regions of capillary dropout, but the highly specific pattern of the retina is altered due to the selective loss of small vessels.

## Anatomy Retinal Vasculature

**Figure 2:**
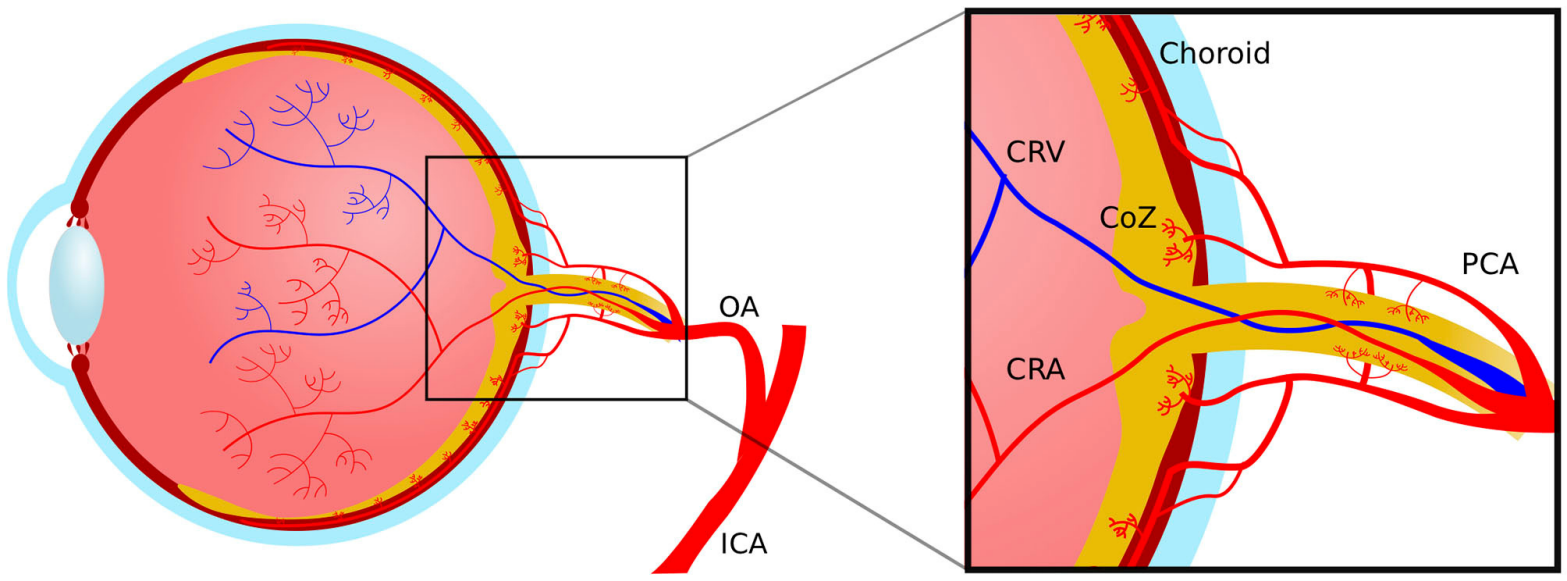
Macrovascular anatomy of the blood supply to the eye. ICA, internal carotid artery; OA, ophthalmic artery; PCA, posterior choroidal artery; CRV, central retinal vein; CRA, central retinal artery; CoZ, circle of Zinn.

**Figure 3:**
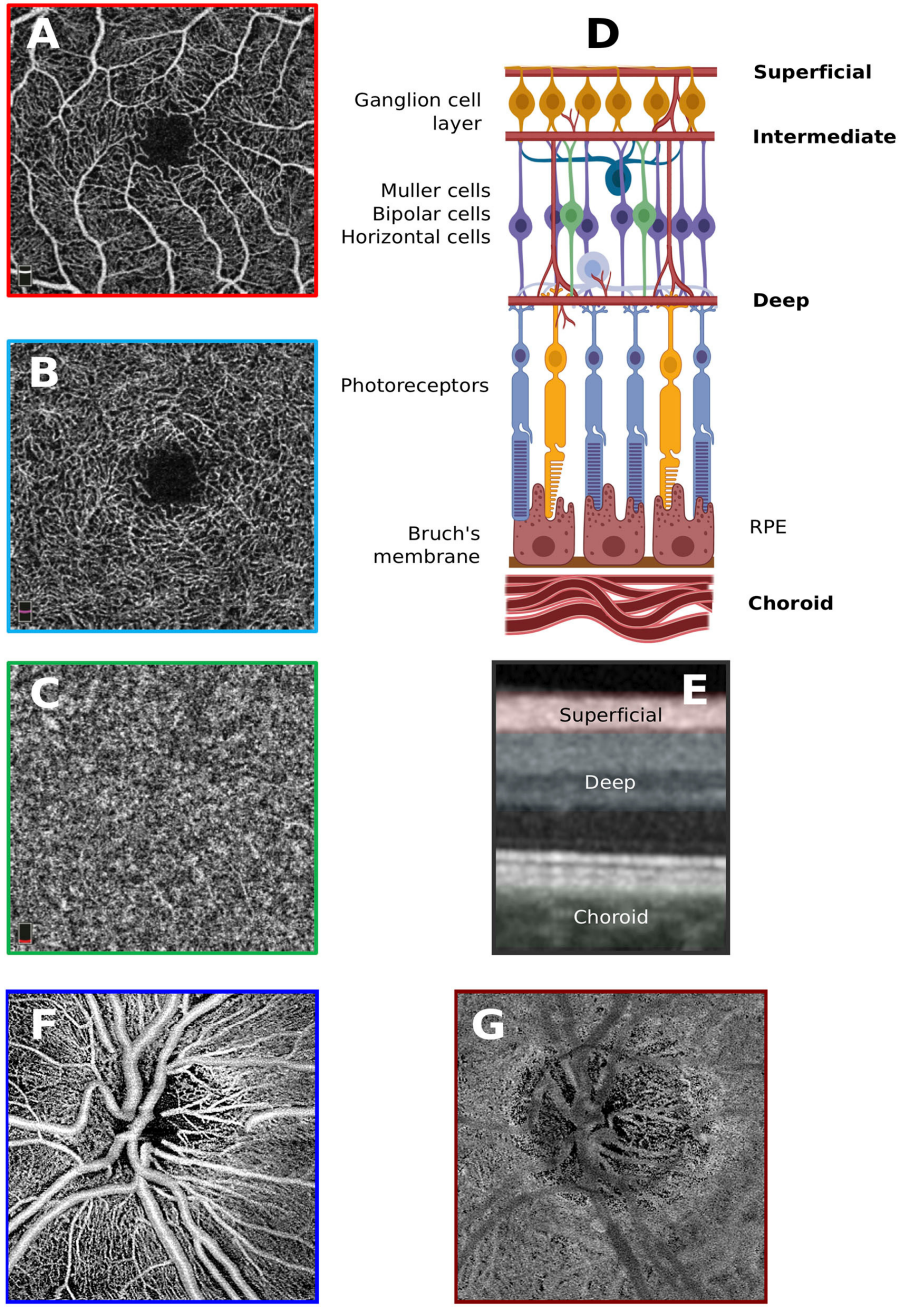
Anatomy of the retinal vascular plexi. **(A)** Macular superficial vascular plexus centered around the fovea. **(B)** Macular deep vascular plexus. **(C)** Macular choroid. **(D)** Locations of the vascular plexi relative to the various cell types that construct the anatomy of the retina. Created with biorender.com. **(E)** Superficial and deep vascular plexi as well as choroid slabs overlain on OCT scan. **(F)** Superficial vascular plexus of the optic nerve head. **(G)** Choroid of the optic nerve head.

Retinal and optic nerve circulation has the challenging goal of supplying nourishment and removing waste without compromising vision. There are two discrete vascular systems in the retina; the retinal and choroidal vessels. Blood supply for both systems comes from the ophthalmic artery, a branch of the internal carotid artery. The ophthalmic artery in turn branches out in two main vessel types that supply the retina, the central retinal artery and the posterior ciliary arteries (Figure 2). The central retinal artery runs with the optic nerve inside the optic nerve sheath and enters the globe through the optic disc after which it branches out to supply the inner retina. The anatomy of the retinal microvasculature plexi is largely determined by constraints that are created by the anatomical organization of different regions in the retina. The three permeating retinal vascular plexi are organized based around the three cellular nuclear layers, the GCL, the inner nuclear layer (INL) and the outer nuclear layer (ONL) (Figure 3). The larger of the plexi, the superficial vascular plexus, mainly supplies the GCL, and roughly is located between the inner limiting membrane and the INL. The intermediate vascular plexus is located around the INL, while the deep vascular plexus is located primarily in the ONL (6, 7). The retinal venous system is interdigitated with the arterial vasculature and venous blood leaves the retina via the central retinal vein.

Retinal capillaries are formed of a layer of endothelial cells encapsulated by a basement membrane in which pericytes are embedded. Retinal capillaries are an important element of the blood-retinal barrier (BRB), the tightly controlled boundary between the intravascular and extravascular environment in the retina (38). Pericytes are essential for contractile function, endothelial barrier integrity and endothelial cell proliferation (38). Fluid and waste are drained from the eye through perivenous spaces, which are part of the ocular glymphatic drainage system (39). Beyond the lamina cribrosa there is no autonomic control of the retinal vasculature, therefore retinal capillaries are dependent on pericytes for local autoregulation of blood flow. Pericytes respond effectively to local tissue needs, and this relationship between local neural activity and changes in blood flow is known as neurovascular coupling. There is an approximate 1:1 ratio of pericytes to endothelial cells allowing for specific neurovascular coupling (40). The basement membrane between pericytes and endothelial cells is also very thin, allowing for increased communication between the two.

In the most common anatomical variant there are typically two posterior ciliary artery branches that run in parallel, but outside of the optic nerve sheath within the orbit. The posterior ciliary arteries supply the choroidal vascular plexus, which receives ~65–85% of the total ocular blood flow. The choroid is responsible for the oxygen supply of the outermost retinal layers, particularly the photoreceptors and retinal pigment epithelium. The choroid is also the source of oxygen for the foveal avascular zone. Finally, the posterior ciliary arteries give rise to the capillary network supplying the exterior optic nerve head in the annulus of Zinn (6, 7).

## OCTA in Neuroinflammatory Disease

### Multiple Sclerosis

#### Vascular Pathology and MS

MS is an immune mediated demyelinating disease of the CNS and is one of the most common neurological causes for permanent disability in young adults (41, 42). Although many advances have been made in the field of MS over the last years, its pathophysiology has not yet been fully elucidated. Recently, increasing evidence indicates that energy failure, due to hypoxia and hypoperfusion, may be an important causal factor in MS. Many studies have demonstrated the presence of hypoxia and hypoperfusion in the CNS of MS affected individuals clinically, radiologically, and histologically (1, 10). For example, cerebral type-III MS lesions histologically frequently resemble hypoxic insult and MS lesions tend to form in watershed areas of the areas of the brain, suggesting a role for hypoxia in its pathophysiology (18, 43). Several studies found a reduction in cerebral blood flow in MS affected individuals, even in the absence of structural damage (44, 45). Cerebral circulation times are increased from a mean of 2.8 s in controls to 4.9 s in all types of MS (46). Hypoperfusion in MS can be identified from very early in its disease course, as perfusion rates in patients with clinically isolated syndrome (CIS) have been found to be reduced (47). Additionally, a study which used near-infrared spectroscopy (NIRS) showed that almost half of MS patients had hemoglobin saturation values that were significantly reduced compared with healthy controls (48). Anemia more than doubles the risk of developing MS and also doubles the risk of experiencing a relapse in MS (49). Furthermore, patients with MS have an increased risk of ischaemic heart disease and stroke, suggesting further susceptibility to microvascular damage (50).

Interestingly, one exploratory case study found evidence for transient reduced blood flow focally in the optic nerve during acute MSON (51). Preliminary data suggests that blood flow in the retinal microcirculation is reduced in patients with relapsing-remitting MS compared with healthy controls (52, 53).

Animal work with experimental autoimmune encephalomyelitis (EAE) revealed that the inflamed spinal cord of affected animals was indeed severely hypoxic and hypoperfused (54–57). The level of hypoxia was temporally and spatially related to the clinical deficit, and predicted subsequent demyelinating damage. Investigations indicated that the hypoxia was caused by insufficient perfusion of the CNS, creating a shortage of oxygen delivery in the inflamed area (54). Accordingly, it was found that therapeutic approaches aimed at alleviating hypoxia, with inspired oxygen, or alleviating hypoperfusion, with vasodilating agents, were related to improved neurologic outcome and reduced later stage demyelination in the affected animals (17). These findings suggest that hypoxia and hypoperfusion may render nerve cells inexcitable through depolarization, causing disability in the acute stage, but also damage oligodendrocytes, causing subsequent demyelination.

Although hypoperfusion is increasingly recognized as a possible important component in the multifactorial etiology of MS, interpretation of its role is controversial. Ambiguity remains if it is a primary process, representing a causal factor in the disease mechanism, or a secondary process, representing the result of lower metabolic demand in atrophied tissue (1). As many existing methods for investigating CNS perfusion are invasive, time-consuming, or laborious, little follow-up data is available to discern the temporal patterns of vascular changes in neuroinflammatory disease. Combining OCT and OCTA may offer ways to explore temporal patterns in vascular changes and structural atrophy of the retinal layers, which might provide important insights into the pathophysiological processes that underlie MS.

#### OCTA in MS

With OCTA being a relatively novel technique, there is a high level of variability in choice of outcome metrics and OCTA system used in studies investigating the microvasculature in MS. Although the variability in test metrics and OCTA systems makes it difficult to make direct comparisons between studies, all currently published studies found a significant reduction in retinal vessel density, in the macular area, the peripapillary area, or both, in MS patients compared with controls. Most also identified clear correlations between structural retinal layer thickness OCT measurements and vessel density (20–22, 58, 59).

The largest study cohort to date consists of 111 RRMS or high-risk CIS patients, who were compared to 50 healthy controls using a Heidelberg Spectralis OCTA (20). This study focused on the macular microvasculature, and found a significant reduction in SVP vessel density from 29.1% in controls to 24.1% in MS eyes (*p* < 0.001), while the DVP was unaffected. Furthermore, MS eyes affected by ON (MSON) had a lower SVP vessel density at 21.7% compared with non-ON (MSNON) eyes at 26.0% (*p* < 0.001). Finally, the difference between vessel densities in MSNON eyes and control eyes just reached significance (*p* = 0.03). Reduced SVP density was related to combined GCL and inner plexiform layer (GCIPL) and pRNFL layer atrophy, while lower DVP densities were related to GCIPL and inner nuclear layer thickness. All analyses were adjusted for age, sex, ON history and within-subject inter-eye correlations. Interestingly, these changes were evident in a cohort of relatively early stage MS, with a mean EDSS scores of 1.5.

**Figure 4:**
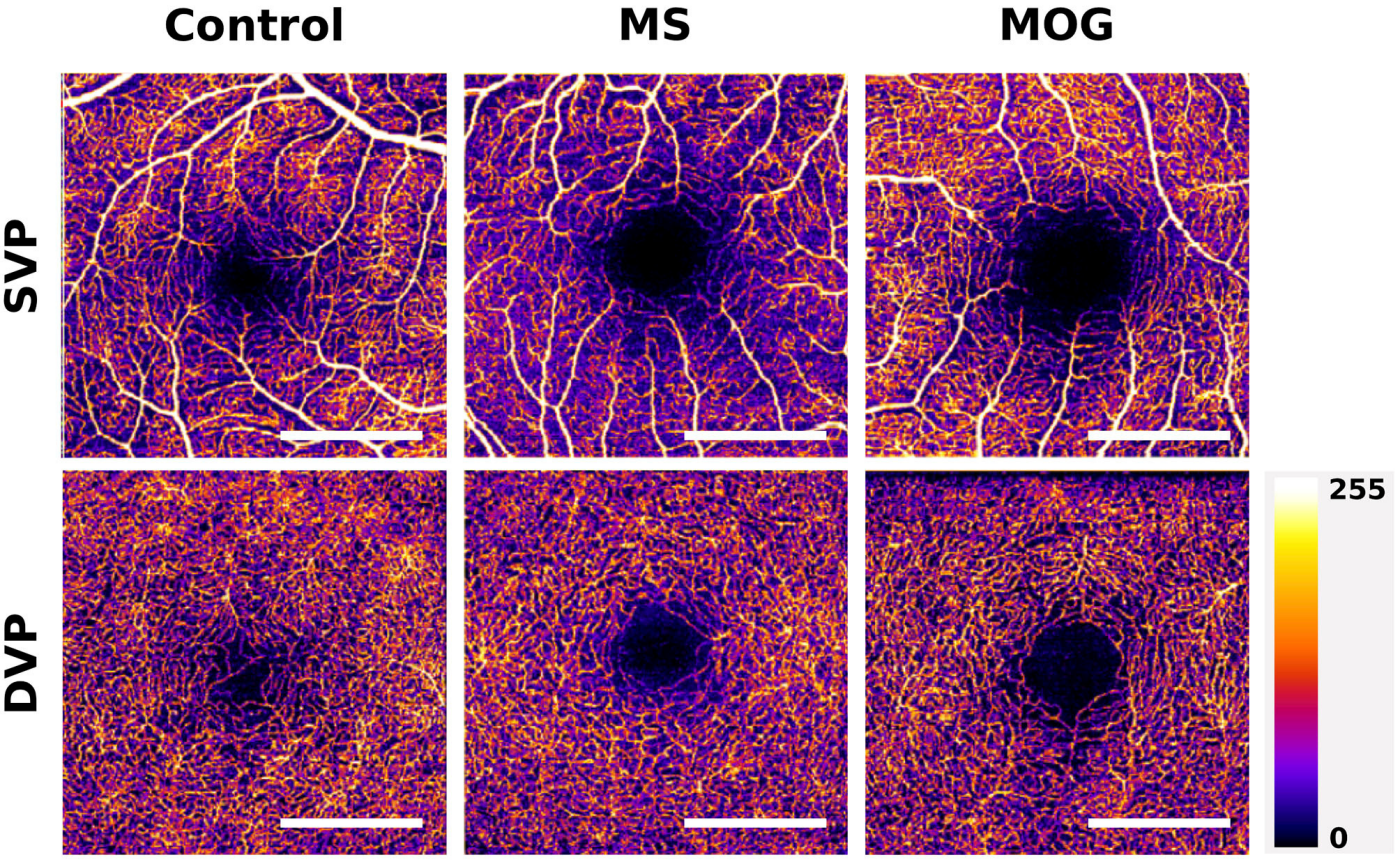
Example of macular superficial vascular plexus (SVP) and deep vascular plexus (DVP) changes in MS and MOG-Ab seropositive NMOSD compared with a healthy control. The scans of MS and MOG-Ab seropositive NMOSD patients are made from eyes without prior optic neuritis. The foveal avascular zones (FAZ) of the MS and NMOSD eyes are greater than these in the healthy control. Scale bar: 1 mm. Scans are 3x3 mm and obtained with the Heidelberg Engineering OCT2 system.

These results are corroborated by several smaller studies that recruited 20 to 50 MS patients and similar numbers of healthy controls, that identified reduced macular SVP vessel densities in eyes of MS patients compared with controls (21, 22, 58, 60), with a significant reduction in SVP vessel density in MSON compared with MSNON eyes being identified in the two larger cohort (21, 60). Two studies identified an additional significant reduction in DVP vessel density, one in MS compared with controls (21) and one only in MSON compared with MSNON (22) (Figure 4). However, not all studies have replicated the finding of reduced macular SVP vessel density in MS (61).

In two smaller cohorts of 35 and 45 patients with relatively more advanced MS, vessel density around the optic nerve head (ONH) was reduced in MS eyes compared to control eyes, as well as in MSON eyes compared with MSNON (61, 62). One study identified reduced vessel densities specifically in the inferior and nasal sections around the ONH in MS patients compared with controls (58), while another study found reductions only in the temporal zone (21). The finding of reduced vessel densities around the ONH has been classified as “capillary dropout” (61).

A recent study developed a novel metric, called volumetric vessel density (VVD), by dividing the macular vessel densities of the SVP and DVP by the tissue volume of the corresponding retinal layers (59). In a large cohort of 80 RRMS patients and 99 matched controls, the VVD of the DVP was higher in MS patients compared with controls. Additionally, the VVD of the DVP and SVP of MSON eyes were significantly higher compared with MSNON eyes (59). This is an interesting new metric that combines structural and microvascular changes in the retina. However, one could argue that it might be clearer to estimate structural and microvascular measures independently, as the VVD is determined to a high degree by changes in retinal thickness. Therefore, the observed increase of the VVD might still reflect a strong decrease in vascular structure density that is obscured by a more pronounced decrease in retinal thickness. This seems not unlikely, as the volume of vascular structures is smaller compared with their corresponding retinal layers and structural retinal atrophy is severe after MSON. This appears to be affirmed by the fact that VVD measures were strongly and negatively correlated with analyzed tissue volumes. However, the VVD is an important step toward incorporating data as we look at the full integrated structural and functional system going forward.

The size and shape of the foveal avascular zone, readily visible using OCTA, has been used as an outcome metric in studies of diabetic retinopathy (63). The foveal avascular zone (FAZ) was used as the outcome metric in one study of MS, and found that neither the area nor perimeter of the FAZ differed between MS patients and healthy controls, even though the FAZ size was negatively related to the vessel density of the SVP and DVP (21).

OCTA data captured in the acute stages of ON is currently lacking, although one small case series of seven MSON patients with good clinical recovery did find significantly reduced SVP macular and ONH vessel densities in affected compared with unaffected eyes 2–8 months after the episode (64). These changes in vessel density were in all cases accompanied by structural thickness loss of the macular GCL layers. Prospective studies investigating the hyperacute stages of MSON might be able to delineate the temporal relationships between structural and vascular changes, which could further the understanding of the role of hypoperfusion in the pathophysiology of MS. The challenge here however will be disc swelling which limts OCTA signal strength.

Just one study has collected longitudinal OCTA data in MS patients to date. One-year follow-up OCTA scans were performed in a cohort of 50 MS patients (mean EDSS 3.5 and average disease duration of 11 years) who had stable disease and were on disease modifying treatment. Interestingly, parafoveal vessel densities increased slightly, a finding just reaching significance (*p* = 0.035), while structural thickness of the pRNFL and GCL remained stable (65). This provides some evidence for possible vascular regeneration or increase of lumen thickness in chronic disease, but additional longitudinal data on the microvasculature in MS, including in its earlier stages, are needed to interpret these findings. Future longitudinal data are required to identify if OCTA can function as a biomarker to predict and monitor disease progression in MS and other neurodegenerative disease (66).

Finally, although glaucoma research suggests that vascular density loss is faster in early-stage disease and has a lower floor-effect in late-stage disease compared with common structural OCT measurements, this has not yet been demonstrated in studies of MS patients (23, 25, 26).

#### Correlation OCTA With Clinical Measures in MS

A lower vessel density of the macular SVP was found to be related to a higher EDSS score and lower low-contrast visual acuity in MS patients (20). Interestingly, in this study results of other clinical outcomes like the 9-hole peg test, the timed 25-foot walk test and the multiple sclerosis functional composite (MSFC) were related to macular SVP vessel density, while combined GCL and inner plexiform layer thickness was not. Another study describes an inverse correlation between visual evoked potential latency times and vessel density of the SVP and DVP (21). Additional, smaller, studies identify no relationship between vascular metrics and clinical or visual outcomes (62). Interestingly, increased vessel density of the macular choriocapillaris has been found to be associated with higher inflammatory disease activity prior to the OCTA examination, suggesting another possible OCTA biomarker in MS (22). Finally, VVD of the macular SVP was positively related to EDSS and disease duration, while being negatively related to low-contrast letter acuity (59).

### Neuromyelitis Optica Spectrum Disorder (NMOSD)

#### Vascular Pathology and NMOSD

The autoimmune inflammatory conditions of NMOSD share cardinal features of ON, longitudinally extensive transverse myelitis and area postrema syndrome that are often relapsing (12, 67). Although attention for vascular changes in neuroinflammatory disease has been primarily focused on MS, there is also evidence that NMOSD pathophysiology is in part vascularly mediated (68, 69). In most cases NMOSD is caused by the pathogenic effects of auto-antibodies targeting the water channel protein aquaporin-4 (AQP4), for which the majority of patients test positive. As AQP4 is predominantly expressed on perivascular astrocytic foot processes in the spinal cord and by Müller cells in the retina, NMOSD has a predilection for the anterior visual system and the spinal cord (70). Approximately 40% of NMOSD patients that test negative for AQP4 antibodies, test positive for antibodies against myelin oligodendrocyte glycoprotein (MOG) (67).

Neuropathology of AQP4-antibody seropositive NMOSD lesions show a perivascular pattern (71, 72), with rim deposits of activated complement components and macrophages found around thickened, fibrosed, and hyalinised vessels (73). Active spinal cord lesions may have an increased density of vascular structures (71). Similar to MS lesions predominantly forming in watershed areas of the brain with poor perfusion, AQP4-Ab seropositive NMOSD lesions preferentially form in the hypo-perfused posterior and lateral spinal columns (74). Interestingly, the choroid plexus expresses AQP4 to a high degree, and immunoreactivity of choroidal plexus epithelial cells is profoundly affected or even lost in NMOSD, even though the choroidal plexus remained structurally intact in neuropathological investigation (75). AQP4 is expressed in a vasculocentric pattern in the optic nerve and spinal cord (72).

Importantly, the predominantly perivascular location of is one of the key neuropathological features that can distinguish AQP4-antibody associated NMOSD from MS lesions at autopsy (72). One study found that retinal vascular changes identified through fundoscopy, such as attenuation of the peripapillary vascular tree (present in 3/40 MS eyes and 22/32 NMOSD eyes; *p* = 0.001) and focal arteriolar narrowing (present in 0/40 MS eyes and 9/32 NMOSD eyes; *p* < 0.001) could successfully distinguish NMOSD from MS patients (19). OCTA may be diagnostically useful tool capable of picking up these vascular differences more easily and systematically. To become clinically useful the aim will be to get high diagnostic sensitivity and specificity levels.

Neuropathological studies of CNS lesions in MOG-antibody seropositive patients reveal distinctly different features compared with AQP4-antibody associated lesions. Although MOG associated lesions have a predilection for perivascular locations, this is less profound compared with AQP4-associated pathology. MOG lesions were found to share pathological characteristics with immunotype II and III MS lesions (76). Type III pattern of demyelination shows similar tissue changes as observed in early stage white matter ischemia, and is therefore hypothesized to potentially be caused in part by hypoxia (77).

#### OCTA in NMOSD

Although there is currently a very limited number of studies published on OCTA in NMOSD, all available data suggest profound decreases in microvascular densities in affected individuals exist (27, 78, 79). A recent study investigating differences in structural thickness and microvascular retinal density of 27 AQP4-antibody seropositive NMOSD patients and 31 healthy controls found a significant difference in vessel density of the peripapillary region and the macular SVP between patients with NMOSD and controls. Interestingly, there were also significant reductions in vessel density in the macular and ONH region when comparing NMOSD patient without previous ON to controls (*p* = 0.023 and *p* = 0.029), while retinal thickness measures of the macular GCL and pRNFL were similar in these groups (27). These findings were replicated by a second study, that also found that vascular densities in the macula and ONH were reduced in NMOSD patients without ON compared with healthy controls, while pRNFL and macular GCL were not (78). This suggests OCTA may be more sensitive to ON-independent damage to the anterior visual system in NMOSD compared with structural OCT (Figure 4). Additionally, these findings of vascular dropout in the absence of structural thinning argue against the hypothesis that reductions in microvascular density are a result of reduced demand in atrophied tissue and, if replicated, may provide evidence for a role of microvascular dysfunction as an independent disease process in NMOSD.

Both the densities of the macular SVP and DVP have been found to be negatively correlated to visual acuity measures in NMOSD (79).

Given that neuropathological research suggests that vascular vessel walls seem to be affected by fibrosis and thickening in NMOSD, it is important to consider that OCTA depicts as a vessel is actually moving blood within a vascular lumen. Therefore, OCTA can depict vessel thinning and capillary dropout, but not the unmoving vessel wall itself. In order to visualize these changes *in-vivo*, devices capable of high-resolution imaging which are not dependent on motion contrast are required, such as adaptive optics coupled ophthalmoscopy. The potentially more profound vascular changes in the anterior visual system of NMOSD patients compared with MS might be the result of the reduced metabolic demand associated with more severe structural atrophy in NMOSD related ON, although the reduction in vascular density in the absence of atrophy argue against this. Furthermore, the vascular pathology might be a result of more mechanical constriction at the ONH due to more severe papilledema during ON. Although there is some evidence for more profound papilledema in ON associated with NMOSD compared with MS, the fact that vascular density reductions are also identifiable in patients without ON is incongruous.

Data on OCTA alterations observed in MOG associated disease and in MOG associated ON specifically is currently lacking. Finally, future research is needed to investigate if certain distinctive qualitative or quantitative differences in retinal microvascular changes exist between MS and NMOSD that might provide valuable additions to the diagnostic arsenal.

## Practical Sources of Bias

There are several important practical factors that should be taken into account when designing an OCTA study, as these may influence outcome metrics and thereby confound findings (23). First, there are various demographic factors that are associated with differences in OCTA results. For example, older age is associated with reduced macular and peripapillary vessel density (80, 81). Furthermore, in glaucoma research, eyes of people from European descent have been identified to have lower vessel density measures compared with eyes of people from African descent (23). Both hypertension and diabetes mellitus are related with decreased macular vessel density, also in the absence of a retinopathy. Chronic use of topical beta-blockers may lead to some reduction in macular SVP density (81).

Myopia is another important factor that is related to significant reductions in vessel density measurements. Highly myopic individuals have been found to have significantly lower vessel densities in the peripapillary region compared with emmetropic eyes. Myopic glaucomatous eyes have more severely reduced vessel densities compared with emmetropic glaucomatous eyes (82, 83). Image magnification in myopic eyes may be in part responsible for these effects (23). On the other hand, a greater disc size was not related to changes in peripapillary vessel density in one cross-sectional study (84).

Besides these demographic factors, there are also some important considerations regarding the timing of OCTA measurements. One study reported that in a small cohort of 13 healthy subjects, vessel density decreased significantly after strenuous physical activity (85). Retinal vessel calibers vary slightly with the cardiac cycle (86). Preliminary data suggests that increases in heart rate and blood pressure are related to higher macular SVP density measurements (87). The same study showed no important diurnal changes in OCTA vessel density.

Interestingly, multiple studies have found that vessel densities were reduced in OCTA scans with lower signal strength (84, 85, 87, 88). This is important since low image quality and motion artifacts are common in OCTA, mainly due to the relatively long acquisition time. As the OCTA signal is based on a threshold of change (movement) detected across the volume, when signal is reduced, vessels are harder to detect leading to reduced vessel density in poor scan quality volumes. Small pupillary size can interfere with image acquisition and generally pupil dilation is necessary for high quality images (89). Finally, media opacities in the lens or especially in the vitreous, can significantly reduce image quality.

Glaucoma research has shown that intra- and intervisit variability of OCTA vessel density measures is between 2.5 and 6.6% in the peripapillary region and between 3.4 and 5.6% in the macular SVP (88, 90). This intra- and intervisit variability of vessel density measures is more than is reported for OCT structural measures of the pRNFL and GCL thicknesses, which is ~1.5%. Importantly, glaucoma affected eyes had worse intervisit repeatability compared with healthy eyes, an effect that can be expected to occur in ON affected eyes as well due to problems with focusing (91). Future developments improving image acquisition speeds and eye-tracking of commercial OCTA systems are needed to bring intra- and intervisit repeatability into a range closer to OCT.

## Advantages and Disadvantages of OCTA

There are opportunities as well as obstacles when using OCTA in neuroinflammatory disease. Compared to other types of retinal vascular imaging, such as fluorescein angiography, OCTA is quick and non-invasive, with no intravenous dyes required. Most devices are user-friendly and require limited training to perform standard imaging. The speed at which OCTA can be obtained means it could be easily integrated into a clinical setting, especially where OCT is already performed and a multimodal retinal imaging setup is available.

The real strength of OCTA is in its multifunctional capacity. As it uses the change in the structural OCT images to map blood flow, both forms of imaging are collected simultaneously. Some studies have combined the information on tissue structure and vascular density to elucidate the vascular impact of multiple sclerosis, increasing diagnostic accuracy. Coupling structural information from retinal thicknesses with vascular density allows for a more complete picture of individual patients, which is highly useful especially in a disease with such variable presentation as MS.

OCTA also has certain disadvantages, namely imaging artifacts and inability to detect dynamic vessel related changes such as leakage or venous sheathing (92). There are yet no consensus quality control criteria similar to what has been achieved for OCT (30). Additionally, OCTA creates a binary 3D figure of moving components to static retinal tissue, but it cannot quantify retinal perfusion as it cannot measure blood velocity. It does not differentiate between areas of high and low flow. Multi-modal imaging approaches which combine Doppler OCT and OCTA are able to add this information, by adding velocimetry to the depth encoded OCTA image (93). In addition, novel high dynamic range OCTA systems are a promising extension to the technology that are sensitive to flow speeds, and are not compromised by angle issues present in Doppler OCT systems (94).

Imaging artifacts are a well-documented limitation to OCTA (95) which are further compounded by visual loss and nystagmus in neuroinflammatory disease (96). Small movements can have profound effects on an OCTA image. As the laser is scanned across the retina, small transverse motions of the eye in respect to that laser can lead to mass displacement of locations in the resulting image, causing a motion artifact. In order to choose the correct location for OCTA imaging, patients are required to fixate on a small target either internal or external to the imaging device. Patients with a history of ON may have poor fixation and are therefore more prone to these motion artifacts. These problems are enhanced in acute ON, where impact on the retinal vasculature may be most profound. Small eye movements can be compounded by larger movements caused by postural difficulties, especially in situations where the standard OCT set up is uncomfortable for the patient. Adjustments can be made but unfortunately there is not yet a handheld OCTA device commercially available to allow for complete accessibility for children and less mobile patients, especially in the later stages of disease. Fast and efficient scanning of the retina to avoid making patients sit or fixate for too long could rectify this issue of motion artifacts.

Segmentation of vascular layers, allowing the user to distinguish individual vessel networks, is a unique ability of OCTA. However, automated segmentation by the device is not always accurate, leading to segmentation artifacts in images which can appear as vessel dropout. Initial identification of the artifact is crucial, and segmentation can be manually adjusted by the user, but there is no consensus currently as to how these artifacts should be identified and remedied.

Ocular complications of MS can also lead to image artifacts. Media opacity, as found in uveitis, greatly diminishes the quality of imaging possible using OCTA. Changes in the optical pathway, such as that caused by microcystic macular oedema (MMO) that occur in MS and NMOSD, could also lead to distortions in the resulting signal.

Overall; motion artifacts, segmentation artifacts and opacity issues can have a profound impact in image quality in OCTA. The importance of quality control of OCT images has been widely accepted in recent times, as evidenced by the OSCAR-IB criteria being integrated into many clinical trials (97–99). Quality control of OCTA images is essential and should be stressed in both the design and interpretation of studies using OCTA in MS. The creation of a set of quality control criteria for OCTA may be very helpful for the standardization of methods between different studies.

**Figure 5:**
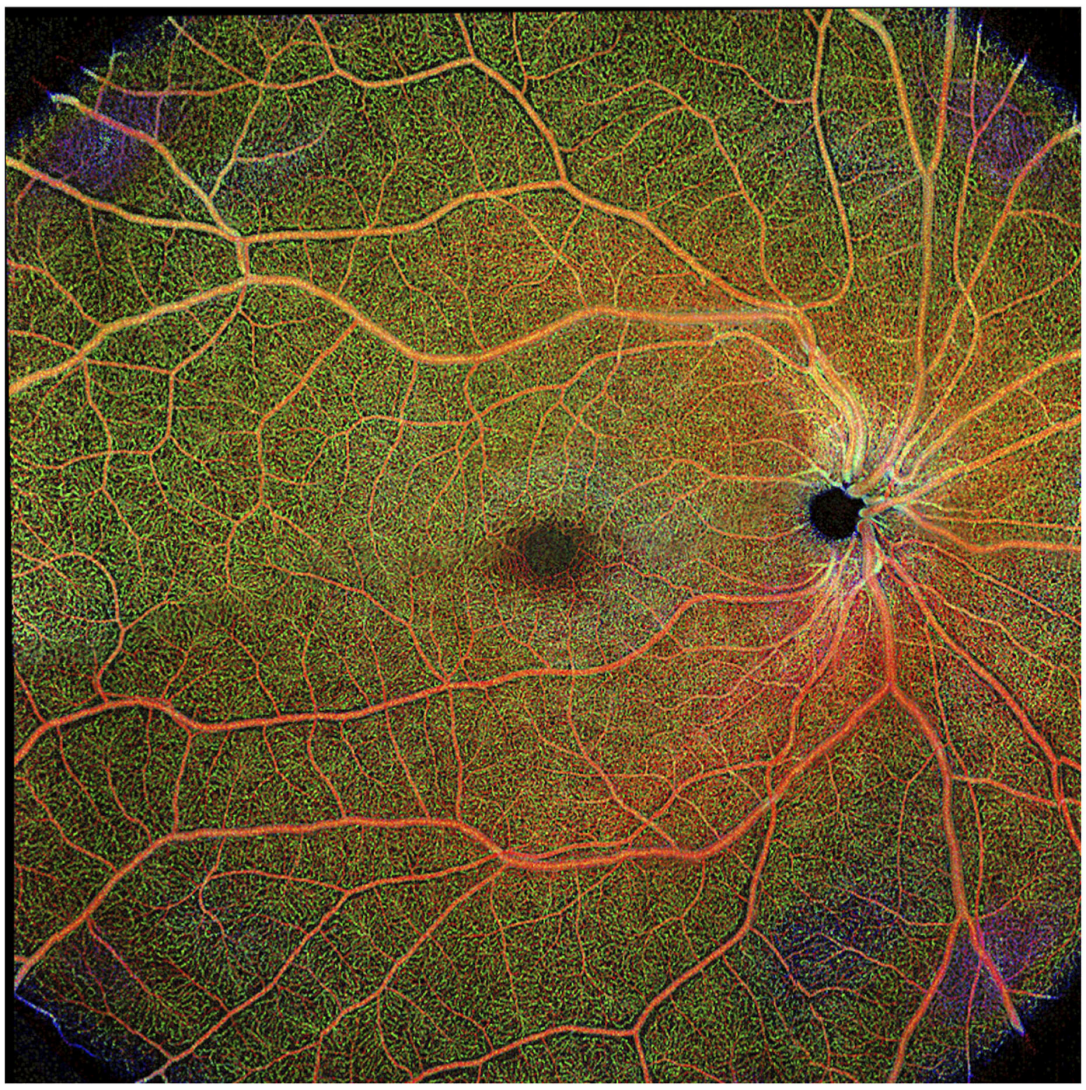
Composite of wide-field optical coherence tomography angiography (OCTA) scan in healthy eye. Scan sample obtained with the Plex Elite 9000 (Carl Zeiss Meditec). Figure is a reprint from Kleerekooper et al. (10).

The field of view available on OCTA devices is limited, with the largest scanning area available using commercial OCTA is 8x8 mm, a field of view of ~30 degrees. Images may be collected separately and “stitched” together, but this extends the image collection time and this montaging capability is not available on all devices (Figure 5). Peripheral changes in the vasculature, such as those caused by vasculitis, are not likely to be detected using even the largest available field of view. Wide-field OCTA when available would be of great benefit to studying the retinal microvasculature in MS.

Each device has its own machine-specific algorithm to measure the difference between B-scans in order to build a picture of the retinal vessels with specific detection thresholds. Many instances where blood flow is altered, such as in leaking vessels, microaneurysms, and polypoidal lesions, will not be detected since they are below this threshold. Interesting vascular features that occur in neuroinflammatory disease, such as venous sheathing or vascular leakage, will not be picked up by OCTA.

## Future Outlook

As discussed in this review, OCTA has only just started to be utilized in research into neuroinflammatory disease. Preliminary studies suggest that the microvascular network in the macula and the peripapillary area is less dense in MS patients, and while prior MSON insult exacerbates this finding it is also present in the absence of MSON. Similar findings are identified in the first studies reporting on AQP4-antibody seropositive NMOSD patients. Interestingly, reduced vascular densities are even identified prior to structural atrophy of retinal layers in NMOSD. Larger cohorts of ON patients in the acute stage are necessary to elucidate the timing of vascular relative to structural changes, to identify if OCTA is indeed more sensitive to early-stage damage and to find if disparities in timing of these two factors may be of diagnostic value in differentiating NMOSD and MS related ON. Furthermore, glaucoma research shows that OCTA has a lower “floor-effect” compared with OCT, being able to pick up further damage in a severely structurally atrophied retina (23–26). If research in neuroinflammatory disease could identify a similar effect, OCTA vascular densities may be more suitable outcome measures in clinical trials where participants have advanced (progressive) disease or have suffered retinal insult due to prior ON compared with OCT. Additionally, choroidal density measures may be a valuable biomarker for disease activity.

The biggest cohort to date studying OCTA in MS (20) found that vascular density correlated with more clinical outcome measures compared with structural OCT metrics. This seems to suggest that the association between certain clinical parameters may be stronger with OCTA compared with OCT thickness measures, which is in line with results in glaucoma research (23). More data is necessary to see if OCTA may provide a more relevant outcome metric that is just as easily obtainable as OCT.

As mentioned before, the possibility to elucidate the timing of vascular relative to structural insult to the retina after acute ON may help to understand the role of hypoperfusion and energy failure in MS pathophysiology. If vascular damage occurs before structural atrophy can be identified, this would give argument to the important role of hypoperfusion in MS related damage. However, if vascular density loss occurs after thinning of retinal layers, this suggests that vascular supply may be reduced due to lower local metabolic demand. This is an important question, given the potential therapeutic possibilities that may reduce MS disability accrual by alleviating hypoperfusion, that OCTA might help answer (17).

Most research has focused on the peripapillary vascular layers and the macular SVP and DVP. As alluded to shortly in this review, there may be benefit to looking into the other retinal vascular layers, such as the choroidal layer. One study identified that a more dense choroidal plexus was related to more severe disease activity in the time before, suggesting it may provide an interesting biomarker (22). However, caution needs to be taken when looking at layers situated more deeply, as it is difficult to accurately interpret the meaning of imaged data. As light travels deeper through retinal tissue, it is more distorted and affected by absorption at different levels, producing a less reliable image.

**Figure 6:**
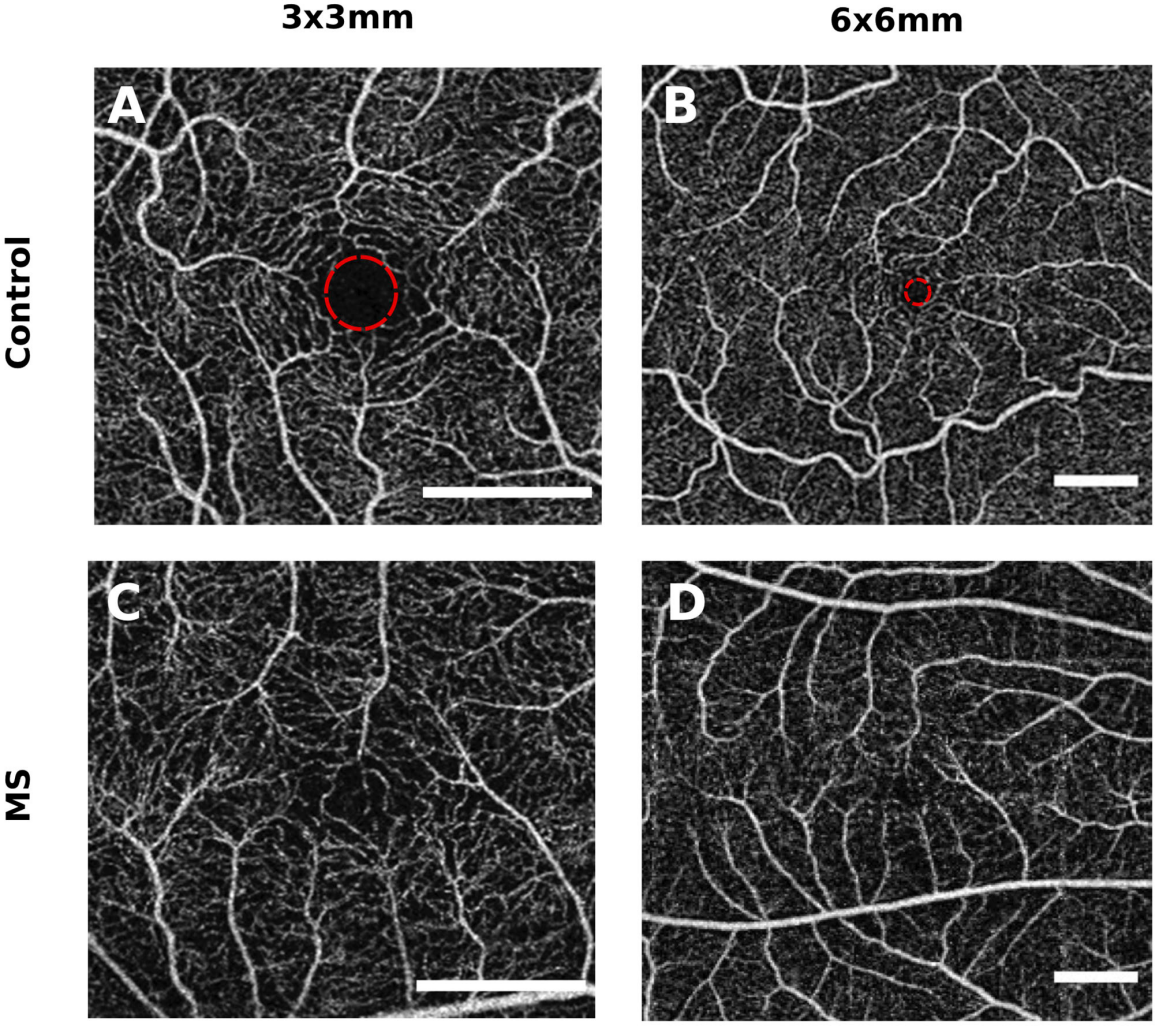
Example of a foveal avascular zone (FAZ) abnormality in a patient with multiple sclerosis (MS). These figures represent an unpublished observation from the authors. However, abnormal FAZ phenotypes are also sometimes observed in healthy subjects, and additional research is necessary to investigate if these are more common in MS compared with healthy controls. **(A,B)** are from two different controls and show a symmetrical and round FAZ. **(C,D)** are from the same MS patient and show an asymmetric FAZ with vessels growing further into the center. Scale bar: 1 mm. The FAZ is delineated with a red circle in **(A,B)** (healthy control eyes).

From personal experience, we know that MS patients may have abnormal phenotypes of the FAZ on OCTA (see Figure 6 for unpublished observations). However, similar FAZ abnormalities are sometimes also observed in healthy controls, and additional study is required to see if these are more common in MS.

Finally, OCTA may be able to pick up temporal and spatial differences in the choroidal blood supply, that is highly adaptive to fluctuations in local demand. Preliminary animal studies have demonstrated spatial and temporal heterogeneity in retinal blood supply (8). OCTA may be used to investigate if neuroinflammatory disease decreases the adaptability of the ocular blood supply.

## Conclusion

OCTA provides an alternative quantitative measure for retinal damage in MS and NMOSD. Early results suggest that in certain circumstances OCTA, compared with OCT, may be more sensitive to retinal changes both early in the disease process, making it useful for detection of disease, and late in the disease process, rendering it suitable for monitoring of disease. Furthermore, it may also serve as a surrogate measure for vascular pathology in the CNS. Preliminary data of patients both with and without optic neuritis consistently reveal lower densities of the retinal microvasculature in both MS and NMOSD compared with healthy controls. Exploring the temporality of vascular relative to structural changes may help answer important questions about the role of hypoperfusion in the pathophysiology of neuroinflammatory disease. Finally, qualitative characteristics of retinal microvasculature may help discriminate between different neuroinflammatory disorders. However, we have to be mindful of issues such as image quality and standardization before incorporating OCTA into clinical practice.

## Author Contributions

IK and SH: literature search, writing main text, and creating figures. AD, ST, and AP: manuscript writing and revision for intellectual content. All authors contributed to the article and approved the submitted version.

## Conflict of Interest

AP reports that the Amsterdam UMC (location VUmc) MS Centre Amsteram and neuro-ophthalmology Expert Centre participated in the OCTIMS trial and the centre has received research support for OCT projects from the Dutch MS Society. The remaining authors declare that the research was conducted in the absence of any commercial or financial relationships that could be construed as a potential conflict of interest.
